# Mobility and HIV vulnerabilities among female sex workers in Guinea-Bissau: findings from an integrated bio-behavioral survey

**DOI:** 10.1186/s12889-023-16744-y

**Published:** 2023-09-25

**Authors:** Emma M. Gorin, Carrie E. Lyons, Brooke A. Jarrett, Mamadu A. Djalo, Kátia Barreto, Fatou M. Drame, Stefan Baral

**Affiliations:** 1 Department of International Health, Johns Hopkins Bloomberg School of Public Health, 615 N Wolfe St, Baltimore, MD 21205 USA; 2 Present address: Department of Environmental Health Sciences, Mailman School of Public Health, Columbia University, 722 W 168th St, New York, NY 10032 USA; 3 Department of Epidemiology, Johns Hopkins Bloomberg School of Public Health, 615 N Wolfe St, Baltimore, MD 21205 USA; 4 ENDA Santé, CP 1041, Bissau, Guinea-Bissau

**Keywords:** Mobility, HIV, Female Sex Workers (FSW), Guinea-Bissau, Respondent-Driven Sampling (RDS)

## Abstract

**Background:**

Mobility is an important risk determinant for HIV given the potential for intermittent access to HIV services. Mobility may be particularly relevant among female sex workers, (FSW) who have been shown to be at high risk for HIV in settings around the world. Data regarding the role mobility plays in exacerbating HIV risks among FSW across Sub-Saharan Africa remains limited, and data on FSW in Guinea-Bissau is sparse.

**Methods:**

FSW in four regions of Guinea-Bissau were recruited with a respondent-driven sampling (RDS) method and participated in an integrated bio-behavioral survey between September 27, 2017 and January 26, 2018. Associations between reported general mobility, mobility to or residence in Bissau, and social and HIV vulnerabilities among FSW in Guinea-Bissau were assessed using multivariable logistic regression models. Population proportions were weighted for RDS sampling, while logistic regression models were not.

**Results:**

Survey respondents included 323 individuals in Bissau, 45 in Bissorã, 140 in Bafatá, and 59 in Gabu. Statistical analyses demonstrated that mobility to more than one destination was significantly associated with recent sex without a condom (ie, sex without a condom within the last three sex acts) with both clients (aOR: 2.47 (95% CI: 1.08, 5.64)) and non-paying partners (aOR: 5.39 (95% CI: 2.61, 11.15)) compared to non-mobility. However, mobility to one or more locations was also associated with higher odds of receiving HIV prevention information, and mobility to more than one location was associated with participating in programming with HIV-related organizations.

**Conclusions:**

These results suggest that while some prevention services including HIV prevention information reach mobile FSW in Guinea-Bissau more than their non-mobile counterparts, the higher rates of condomless sex among mobile FSW suggest that HIV prevention needs may remain unmet for mobile FSW in Guinea-Bissau. Additionally, the results suggest a nuanced relationship between mobility, place of residence, and HIV and social vulnerabilities and prevention indicators.

**Supplementary Information:**

The online version contains supplementary material available at 10.1186/s12889-023-16744-y.

## Background

Population mobility and migration have long been considered to be determinants for Human Immunodeficiency Virus (HIV) transmission [1–3]. However, mobility remains inconsistently defined in the literature [4, 5]. Broadly, it is understood to include short term, temporary and seasonal population movement, and is sometimes defined as involving at least one overnight stay [3–6]. Some definitions of mobility also encompass migration, which is establishing residence or staying for an extended period in a foreign country [3]. In recent decades, mobility and migration have been reconceptualized not just as means of HIV transmission across regions, but as risk factors for HIV infection among mobile populations [3, 7]. For example, mobility has been observed to be associated with HIV vulnerabilities, including more sexual concurrency [8], new sexual partners [9] and more transactional sex [9]. Prior literature has posited that mobility and migration may contribute to these vulnerabilities because of the social and medical disruptions that occur upon departure from a place as well as the marginalization, discrimination, and lack of awareness of local services experienced while adapting to new, unfamiliar environments [3, 7, 10, 11].

Female sex workers (FSW) are a key population at risk for HIV and are disproportionately impacted by HIV throughout low and middle income countries (LMIC) [12]. This is the case in both generalized and concentrated epidemic settings, as even FSW in high prevalence settings have 12 times the odds of HIV compared to all women of reproductive age [12]. FSW have also been recognized as a highly mobile population in many contexts [1, 13–16]. It has been suggested that sex workers travel in response to shifting demand and to accompany mobile client populations [14, 16]. A systematic review found research on mobility and sex work to be sparse, and results related to mobility and social and HIV vulnerabilities among FSW to be varied [4]. The authors suggested that this is likely due to both inconsistency in defining mobility and its highly context-dependent dynamics [4, 5]. Associations have been observed between mobility and risk factors for HIV, including sexually transmitted infections (STI) symptoms, physical and sexual violence, anal sex with clients, and having sex without a condom for higher pay among sex workers in India [17]; reduced control over condom negotiations and increased violence among FSW in Canada [18]; and increased gender-based violence among FSW in Tanzania [19, 20]. However, increased mobility has also been found to be associated with increased participation in community mobilization activities and increased clinic attendance among FSW in Zimbabwe [21].

Providing HIV prevention and treatment services that are appropriate for highly mobile populations, and especially FSW, is essential globally, including in West and Central Africa where the pooled HIV prevalence among FSW is 34.9% [22]. Evidence on mobility and HIV among FSW in Guinea-Bissau is not currently available in the literature, and information on FSW in Guinea-Bissau generally is limited [23]. The available evidence on FSW in Guinea-Bissau suggests high rates of HIV:according to UNAIDS, Guinea-Bissau reported an HIV prevalence of nearly 40% among FSW as of 2012 [24]. A survey of 440 FSW in seven cities in Guinea-Bissau, which recruited participants using venue-based and peer-referral sampling from 2014 to 2017, found an overall HIV prevalence in the study population of 26.8% [25]. Furthermore, mobility has been found to be a vulnerability and an impediment to HIV care among adults in Guinea-Bissau. For instance, in Caio, a rural community in Guinea-Bissau, mobility was found to be high and was associated with certain risk behaviors, including higher odds of having multiple partners among men and higher odds of having casual partners among women [26]. Interestingly, the same study found mobility to be associated with increased, though not significantly increased, condom use [26]. Mobility has also functioned as an impediment to HIV care in Guinea-Bissau. In a 2017 study, authors found mobility to be one of the main reasons that patients cited for disengagement from HIV care, and mobility could plausibly constitute a challenge for HIV prevention programming as well [27].

Interventions designed to reach FSW are crucial for preventing new HIV infections and transmission among FSW themselves, their clients, and the wider population [28, 29]. To reach mobile FSW specifically, it is also important to characterize patterns of mobility in the FSW population and associated risks. The primary objective of this analysis is to characterize associations between mobility and social and HIV vulnerabilities among FSW in Guinea-Bissau.

## Methods

### Study setting and design

Guinea-Bissau is a country of 1.8 million people that gained independence from Portugal in 1974 [30, 31]. The major urban area is the capital, Bissau, which has a population of over 500,000 [31]. A cross-sectional, integrated bio-behavioral survey (IBBS) was conducted in the regions of Bissau, Gabú, Bafatá, and Oio in Guinea-Bissau in 2017 and 2018 by Enda Santé—Bissau. This study was approved by the Comité Nacional de Ética na Saúde (CNES) da Guiné-Bissau.

### Study population

Participants were recruited in four cities in Guinea-Bissau between September 27, 2017 and January 26, 2018. Individuals were eligible to participate if they earned the majority of their income over the past 12 months from selling sex, were 18 years or older, were assigned the female sex at birth, and had lived primarily in the geographical area of recruitment for at least three months. Participants were recruited using respondent-driven sampling (RDS) [32] and gave consent verbally to protect their identities. Initial peer recruits, known as “seed participants”, were selected by the study team in collaboration with community representatives. After completing study procedures, each seed participant and each subsequent study participant was invited to refer three individuals from their network to participate in the survey, using numbered coupons to track by whom each participant was recruited. Participants were asked to report their network size by answering the following question: *“How many women do you know personally who practice sex work? I.e. you know them and they know you, you have seen them in the last 2 years, and you could contact them if you needed to? Take time to think about it.”* Seed participants were included in all analyses. Details on the RDS sampling approach for each of the four cities are included in Table 1.

**Table 1:** RDS sampling details for surveys of FSW in 2017 and 2018 in Guinea-Bissau (*N* = 567)

City	Number of participants	Number of seeds	Greatest chain length
Bissau	323	8	22
Bissorã	45	3	4
Bafatá	140	3	5
Gabu	59	3	6

### Study measures

Enrolled individuals participated in an interviewer-administered questionnaire, which included questions about demographics (i.e., marital status, children, employment outside of sex work, age, and age of first sex work engagement), social and HIV vulnerabilities (i.e., condom use, access to healthcare, opportunities to learn about HIV prevention, HIV-related knowledge, and stigma), and potential confounders (i.e., city of residence, income, education, place of birth, and previous HIV diagnosis). Mobility was assessed on the basis of overnight stays away from home. Participants were asked: “In the past six months, other than your usual place of residence, in how many different cities/towns have you spent the night?” The exposure of interest was the extent of mobility in the six months prior to the survey, which was divided into three categories: non-mobile, mobile to one location, and mobile to more than one location. Additionally, a variable based on participants’ relationship with Bissau (live elsewhere and do not travel to Bissau, live elsewhere and travelled to Bissau in the last six months, live in Bissau) to address whether services available in Bissau specifically are important for the different experiences of FSW based on mobility. Stigma was categorized according to metrics described by Grosso et al. [33]. To assess recent condom use, participants were asked if a condom was used during each of the last three sex acts with a client and each of the last three sex acts with a non-paying partner. Participants were considered to have had recent condomless sex – assessed separately for clients and non-paying partners – if they reported “no” for any of the most recent three sex acts. Detailed definitions for each variable are included in Supplementary Table 1.

### Statistical analyses

Simple descriptive statistics of the prevalence of mobility, demographics, social and HIV vulnerabilities, and potential confounders were assessed using estimated population percentages for categorical variables adjusted for the RDS sampling approach. Single and multivariable logistic regression models were used to assess associations between the exposure (mobility) and social and HIV vulnerabilities. Previous studies in India [17], Canada [18], Tanzania [19, 20] and Zimbabwe [21], described in the background section above, have observed HIV-related vulnerabilities among mobile FSW related to STIs, physical and sexual violence, condom use, “community mobilization” and healthcare access. Based on this prior evidence, the authors’ hypotheses about the plausibility of association with mobility, and availability and missingness of variables in the dataset, social and HIV vulnerabilities broadly related to condom use, healthcare access, HIV-related knowledge, participation in HIV and FSW community related activities, and stigma were selected to be investigated. Covariates included in each multivariable model included migration, city of residence, education, and self-reported HIV diagnosis, and age. Covariates to be included in each model were selected a priori based on the authors’ reasoning that these variables had the potential to confound the relationship between mobility and the social and HIV vulnerabilities of interest. For instance, migration was included because the authors posited that individuals who had migrated from different countries or regions of Guinea-Bissau may have experienced social vulnerabilities related to migration, and likewise may be more mobile because of mobility to their home country or region, and that migration could therefore confound the association between mobility and social and HIV vulnerabilities. For consistency and comparability across each of the models, the same covariates were included in each model.

Additionally, to address the question of whether residence in or mobility to Bissau specifically was important for the observed trends, single and multivariable logistic regression models were used to assess associations between participants’ relationship with the city of Bissau and the same list of social and HIV vulnerabilities. For this secondary analysis, the exposure of interest was categorized as: live outside Bissau and do not travel to Bissau/live outside Bissau and travel to Bissau/live in Bissau.

An alpha of < 0.05 was used to assess statistical significance for all regression models. Logistic regression models were not weighted for RDS sampling. Complete case analyses were used for all regression models. While covariates were selected a priori, the McFadden pseudo-R^2^ statistic was consulted to assess model fit. Statistical analyses were performed in Stata 15 [34] and population proportions were estimated in R using the RDS package [35].

## Results

### Characteristics of the FSW populations in the four survey sites

Characteristics of the FSW populations in the four surveyed cities, weighted to account for the RDS sampling approach, are included in Table 2. Of the 567 FSW, a majority were surveyed in Bissau (57%; *n* = 323, Table 1). Education varied by city, with substantial proportions of the population in Bissau, Bissorã and Bafatá populations receiving some secondary school, while the majority of the Gabu FSW population did not receive education (70.61%). Most FSW in each city are non-migrants (defined as born within the same state they currently reside), while a substantial number (40.35%) of FSW in Bissau are domestic migrants. Most FSW at all sites had never married and were between the ages of 18–24. Distributions of employment, number of years selling sex, and number of children varied between cities, as did self-reported HIV diagnosis, positive HIV test, and positive hepatitis B test. Notably, there was a higher rate of both self-reported HIV diagnosis (24.16%) and positive HIV test (37.19%) in Gabu than in the other three surveyed cities.

**Table 2:** Description of adult FSW in four cities in 2017 and 2018 in Guinea-Bissau (*N* = 567)

	**Bissau, *N* = 323** **% (95% CI)**	**Bissorã, *N* = 45** **% (95% CI)**	**Bafatá, *N* = 140** **% (95% CI)**	**Gabu, *N* = 59** **% (95% CI)**
*Education*				
None	11.31 (8.35, 14.27)	1.28 (0.37, 2.19)	19.07 (12.94, 25.21)	70.61 (57.7, 83.51)
Quaranic school or some/completed primary school	13 (9.68, 16.32)	10.54 (1.41, 19.66)	34.49 (24.27, 44.7)	18.87 (10.21, 27.54)
Some secondary school	60.47 (52.43, 68.5)	76.21 (62.48, 89.95)	40.82 (30.99, 50.65)	10.14 (0, 20.89)^a^
Completed secondary school or more	15.22 (10.99, 19.45)	11.97 (1.53, 22.41)	5.62 (1.3, 9.95)	0.38 (0.11, 0.65)
*Migration*				
Non-migrant	57.3 (51.01, 63.6)	86.78 (76.66, 96.89)	94.13 (90.95, 97.31)	90.18 (77.5, 102.87)
Domestic migrant	40.35 (34.09, 46.61)	13.22 (3.11, 23.34)	5.71 (2.55, 8.87)	3.84 (0.82, 6.86)
International migrant	2.35 (1.8, 2.89)	0 (0, 0)	0.16 (0.11, 0.21)	5.98 (-6.86, 18.82)
*Marital status*				
Never married	84.31 (79.77, 88.85)	70.25 (18.04, 122.46)	72.21 (62.11, 82.31)	93.77 (90.19, 97.34)
Married or partnered	11.59 (7.02, 16.16)	26.24 (-26.53, 79)	25.53 (15.25, 35.81)	2.07 (0.76, 3.37)
Divorced or widowed	4.1 (3.23, 4.96)	3.51 (-2.27, 9.3)	2.26 (1.63, 2.88)	4.17 (1.53, 6.8)
*Employment outside of sex work*				
No other employment or retired	40.61 (32.7, 48.52)	25.79 (2.97, 48.62)	62.79 (52.99, 72.59)	68.31 (54.96, 81.66)
Student	39.02 (27.57, 50.46)	47.58 (7.78, 87.39)	22.17 (12.87, 31.48)	0 (0, 0)
Other employment	20.37 (15.64, 25.11)	26.62 (7.15, 46.1)	15.04 (9.16, 20.93)	31.69 (18.34, 45.04)
*Age*				
18–24	68.25 (61.84, 74.65)	72.07 (60.35, 83.8)	82.61 (77.32, 87.91)	53.73 (35.1, 72.36)
25–29	16.98 (13.27, 20.7)	24.76 (13.44, 36.09)	13.85 (9.19, 18.51)	18.21 (8.35, 28.07)
30–34	9.11 (6.91, 11.31)	3.16 (0.07, 6.25)	1.25 (0.8, 1.7)	17.39 (7.55, 27.22)
35 +	5.66 (4.32, 7.01)	0 (0, 0)	2.28 (1.53, 3.04)	10.67 (4.89, 16.45)
*Number of years selling sex*				
0 to 2	34.29 (28.59, 39.99)	60.79 (38.69, 82.89)	53.05 (42.83, 63.27)	36.58 (7.66, 65.51)
3 to 5	30.65 (22.72, 38.58)	22.08 (7.99, 36.17)	17.69 (9.46, 25.92)	36.54 (14.97, 58.12)
6 +	35.06 (29.02, 41.11)	17.13 (6.98, 27.28)	29.27 (21.37, 37.16)	26.87 (5.52, 48.22)
*Number of children*				
0	40.11 (29.92, 50.3)	32.1 (13.24, 50.95)	60.21 (51.15, 69.28)	21.47 (0, 53.78)^a^
1 to 2	42.83 (35.15, 50.52)	50.9 (33.29, 68.51)	24.27 (16.45, 32.09)	32.04 (13.94, 50.13)
3 to 5	14.35 (10.52, 18.19)	14.27 (5.42, 23.11)	10.51 (6.46, 14.56)	33.2 (13.67, 52.72)
6 +	2.7 (1.94, 3.46)	2.74 (-3.18, 8.66)	5.01 (3.21, 6.81)	13.3 (5.47, 21.14)
*Self-reported previous HIV diagnosis*	0.89 (0.68, 1.09)	0 (0, 0)	2.02 (1.27, 2.77)	24.16 (10.48, 37.84)
*Tested positive for HIV*	11.67 (9.36, 13.98)	7.19 (0, 15.29)^a^	11.05 (5.39, 16.72)	37.19 (22.47, 51.91)
*Tested positive for hepatitis B*	11.93 (7.43, 16.42)	9.81 (0, 19.72)^a^	13.63 (6.53, 20.74)	9.03 (1.51, 16.54)

Description of the FSW population in each city surveyed. The estimated population percentages have been adjusted to account for the RDS sampling approach

^a^The lower bound of the confidence interval was adjusted to zero for any ranges with bounds estimated below zero

### Social and HIV vulnerabilities in FSW study population

Reported mobility and social and HIV vulnerabilities of the FSW populations in each surveyed city, adjusted for RDS sampling, are summarized in Table 3. Mobility varied across cities, with FSW in Bissau generally being less mobile (62.06% non-mobile) than those in the other three cities. Among the four cities, sex without a condom was highest with both clients (27.07%) and non-paying partners (40.33%) in Bissau. Access to healthcare and STI symptoms varied widely between cities: notably, 71.47% of FSW in Bissorã had ever visited a health center for their own health and 53.91% had experienced STI symptoms in the past 12 months, more than any other city. Very few FSW across all study sites have ever used pre-exposure prophylaxis (PrEP). More than half of FSW in Bissau, Bafatá and Gabu (62.67%, 63.74% and 60.03% respectively) and nearly half (47.15%) in Bissorã had received HIV prevention information in the past six months. FSW in Gabu were more likely to have participated in activities to promote the rights of FSW (29.27%) and to have participated in activities with an HIV-related organization (41.99%). HIV-related knowledge varied widely across surveyed cities. Stigma from family and friends was experienced most in Bissorã (15.65%) while stigma from the police was experienced most in Gabu (10.64%) compared to the other study sites.

**Table 3:** Prevalence of self-reported vulnerabilities among adult FSW in four cities in 2017 and 2018 in Guinea-Bissau (*N* = 567)

	**Bissau, *N* = 323** **% (95% CI)**	**Bissorã, *N* = 45** **% (95% CI)**	**Bafatá, *N* = 140** **% (95% CI)**	**Gabu, *N* = 59** **% (95% CI)**
*Mobility*				
Non-mobile	62.06 (54.51, 69.6)	28.89 (9.61, 48.17)	31.63 (22.3, 40.96)	44.96 (26.98, 62.94)
Mobile to one location	28.57 (23.19, 33.95)	26.41 (11.73, 41.09)	40.75 (30.37, 51.13)	20.52 (10.3, 30.74)
Mobile to more than one location	9.38 (0.24, 18.51)	44.7 (13.03, 76.37)	27.62 (20.69, 34.55)	34.52 (17.42, 51.63)
*Sex without a condom*				
With client(s) (last three sex acts)	27.07 (6.63, 47.52)	8.99 (0, 19.44)^a^	13.33 (5.8, 20.87)	22.83 (0, 66.75)^a^
With non-paying partner(s) (last three sex acts)	40.33 (28.7, 51.97)	22.13 (8.17, 36.08)	17.4 (10.04, 24.76)	15.21 (0, 32)^a^
*Health and healthcare*				
Visited health center for own health (ever)	13.87 (10.45, 17.3)	71.47 (55.39, 87.56)	4.12 (2.79, 5.46)	57.02 (33.49, 80.55)
STI symptom (past 12 months)	9.66 (7.16, 12.16)	53.91 (37.28, 70.54)	16.33 (11.99, 20.68)	30.38 (17.08, 43.69)
Ever taken PrEP	0.67 (0.51, 0.82)	85.42 (53.14, 117.71)	2.39 (1.74, 3.04)	2.01 (0.91, 3.11)
*Opportunities to learn about HIV prevention*				
Received HIV prevention information (past 6 months)	62.67 (55.39, 69.95)	47.15 (30.12, 64.17)	63.74 (52.9, 74.58)	60.03 (43.97, 76.1)
Participated in an activity to promote the rights of FSW (past 6 months)	4.8 (3.81, 5.79)	0 (0, 0)	3.98 (2.45, 5.51)	29.27 (15.41, 43.12)
Participated in an HIV-related organization (past 6 months)	9.42 (7.55, 11.3)	99.09 (98.49, 99.69)	6.62 (4.6, 8.65)	0 (0, 0)
*HIV Knowledge*				
Able to correctly identify anal sex as type of sex with greatest risk of HIV infection	2.17 (1.67, 2.66)	100 (100, 100)	40.35 (30.87, 49.82)	89.52 (35.14, 143.9)
Able to correctly identify water-based lubricant as safest option	4.56 (3.58, 5.55)	8.07 (3.32, 12.82)	0 (0, 0)	100 (100, 100)
Able to correctly state that HIV can be transmitted through sharing needles	18.87 (12.87, 24.86)	87 (79.16, 94.83)	51.74 (41.79, 61.7)	69.3 (47.83, 90.77)
*Stigma*				
Experienced stigma	2.77 (2.11, 3.43)	9.17 (3.2, 15.14)	0.21 (0.14, 0.28)	11.31 (5.09, 17.53)
Anticipated or experienced healthcare stigma	1.45 (1.12, 1.78)	8.21 (0, 19.51)	0 (0, 0)	1.68 (0.73, 2.63)
Stigma from family and friends	0.95 (0.73, 1.17)	15.65 (8.76, 22.55)^a^	0.36 (0.24, 0.48)	3.37 (1.41, 5.33)
Stigma from the police	0.87 (0.67, 1.06)	3.33 (0, 11.79)^a^	0.64 (0.51, 0.77)	10.64 (5.05, 16.23)

Estimated percentages of the FSW population in each surveyed city experiencing the HIV-related vulnerability or factor listed. The population proportions have been adjusted to account for the RDS sampling approach

^a^The lower bound of the confidence interval was adjusted to zero for any ranges with bounds estimated below zero

Reported destinations of mobility among survey participants (locations in which the respondent spent the night away from home) are reported in Table 4. Some participants reported spending the night away from their place of residence but within their city of residence: for the purposes of these analyses, we still considered this type of report to be mobility, with the understanding that even locations within the same city or region can require significant travel time in this context.

**Table 4:** Destinations of mobility among FSW survey participants in four cities in 2017 and 2018 in Guinea-Bissau (*N* = 567)

	Location in which respondent reported spending at least one night away from home						
	**Bissau**	**Bissorã**	**Bafatá**	**Gabu**	**Other domestic destination**	**Senegal**	**Other international destination**
City of residence							
Bissau	53	5	13	3	44	31	7
Bissorã	15	15	0	1	12	1	0
Bafatá	56	0	3	30	78	7	3
Gabu	11	0	0	19	4	4	2

Location in which survey respondent spent the night away from home by city of residence among FSW surveyed in Guinea-Bissau in 2017 and 2018. Some respondents reported multiple destinations, and therefore may be included more than once in this table

### Associations between mobility and HIV and social vulnerabilities among FSW

Adjusted and unadjusted logistic regression models revealed several associations between mobility and social and HIV vulnerabilities (Table 5). Mobility to more than one location was associated with higher odds of condomless sex with clients (aOR: 2.47 (95% CI: 1.08, 5.64)) and non-paying partners (aOR: 5.39 (95% CI: 2.61, 11.15)) compared to non-mobile FSW. Mobility to one location was associated with increased odds of condomless sex with non-paying partners (aOR: 2.85 (95% CI: 1.69, 4.81)), and with decreased odds of condomless with clients, although the association was not significant (aOR: 0.55 (95% CI: 0.25, 1.20).

**Table 5:** Associations between mobility and HIV vulnerabilities/prevention indicators among FSW in four cities in 2017 and 2018 in Guinea-Bissau (*N* = 567)

Outcome/HIV vulnerability	Unadjusted OR (95% CI)*	*P*-value**	Adjusted*** OR (95% CI)*	*P*-value**
*Sex without a condom*				
With a client (last 3 sex acts)				
Mobile to 1 location	0.60 (0.29, 1.25)	0.172	0.55 (0.25, 1.20)	0.132
Mobile to > 1 location	**2.34 (1.14, 4.80)**	0.02	**2.47 (1.08, 5.64)**	0.032
With a non-paying partner (last 3 sex acts)				
Mobile to 1 location	**2.47 (1.51, 4.04)**	< .001	**2.85 (1.69, 4.81)**	< .001
Mobile to > 1 location	**3.13 (1.71, 5.74)**	< .001	**5.39 (2.61, 11.15)**	< .001
*Health and healthcare*				
Visited health center for own health (ever)				
Mobile to 1 location	0.89 (0.60, 1.31)	0.551	0.99 (0.64, 1.53)	0.957
Mobile to > 1 location	1.30 (0.79, 2.13)	0.3	**2.14 (1.11, 4.12)**	0.023
Reported any STI symptoms (past 12 months)				
Mobile to 1 location	1.09 (0.73, 1.63)	0.669	1.10 (0.72, 1.69)	0.661
Mobile to > 1 location	1.24 (0.74, 2.09)	0.411	1.38 (0.76, 2.51)	0.293
*Opportunities to learn about HIV prevention*				
Received info on HIV prevention in (past 6 months)				
Mobile to 1 location	**2.19 (1.50, 3.20)**	< .001	**2.11 (1.42, 3.12)**	< .001
Mobile to > 1 location	**3.23 (1.91, 5.46)**	< .001	**2.84 (1.59, 5.08)**	< .001
Participated in activity to promote the rights of FSW (past 6 months)				
Mobile to 1 location	0.81 (0.44, 1.49)	0.497	0.76 (0.39, 1.48)	0.42
Mobile to > 1 location	0.80 (0.35, 1.81)	0.587	1.03 (0.38, 2.75)	0.959
Participated in HIV prevention organization (past 6 months)				
Mobile to 1 location	**2.44 (1.51, 3.94)**	< .001	**2.88 (1.66, 4.99)**	< .001
Mobile to > 1 location	1.43 (0.74, 2.77)	0.288	1.58 (0.69, 3.63)	0.281
*HIV knowledge*				
Able to correctly identify anal sex as the type of sex with greatest risk of HIV transmission				
Mobile to 1 location	**2.51 (1.47, 4.27)**	0.001	**2.22 (1.17, 4.23)**	0.015
Mobile to > 1 location	1.11 (0.50, 2.46)	0.805	**0.28 (0.10, 0.73)**	0.01
Able to correctly identify water-based lubricant as safest option with condoms				
Mobile to 1 location	1.56 (0.84, 2.88)	0.16	1.59 (0.82, 3.08)	0.17
Mobile to > 1 location	0.70 (0.26, 1.91)	0.488	1.23 (0.36, 4.21)	0.746
Able to correctly state that HIV can be transmitted through sharing needles				
Mobile to 1 location	**1.73 (1.17, 2.56)**	0.006	**1.78 (1.17, 2.70)**	0.007
Mobile to > 1 location	0.66 (0.40, 1.07)	0.09	**0.48 (0.27, 0.85)**	0.012
*Stigma*				
Reported experienced stigma (past 6 months)				
Mobile to 1 location	1.63 (0.92, 2.87)	0.095	1.77 (0.96, 3.28)	0.067
Mobile to > 1 location	0.97 (0.42, 2.23)	0.944	1.49 (0.56, 3.96)	0.427
Reported anticipated or experienced healthcare stigma (past 6 months)				
Mobile to 1 location	1.44 (0.53, 3.91)	0.472	1.64 (0.59, 4.58)	0.342
Mobile to > 1 location	1.19 (0.31, 4.60)	0.798	1.43 (0.32, 6.38)	0.636
Reported stigma from family or friends (past 6 months)				
Mobile to 1 location	1.02 (0.44, 2.34)	0.966	1.02 (0.43, 2.43)	0.959
Mobile to > 1 location	1.38 (0.51, 3.71)	0.521	1.45 (0.47, 4.40)	0.517
Reported stigma from police (past 6 months)				
Mobile to 1 location	1.30 (0.52, 3.25)	0.581	1.36 (0.51, 3.62)	0.54
Mobile to > 1 location	0.95 (0.25, 3.52)	0.935	1.36 (0.31, 5.86)	0.683

Unadjusted and adjusted associations between mobility and self-reported social and HIV vulnerabilities among adult female sex workers recruited via respondent-driven sampling by Enda Santé-Bissau in 2017 and 2018 across Bissau, Bissorã, Bafatá, and Gabu in Guinea Bissau, Africa (*N* = 567)

^*^ Non-mobile is the reference group for all ORs and aORs

^**^ Bold text indicates a significant association at an alpha of < 0.05

^***^ Adjusted for migration, city of residence, education, and self-reported HIV diagnosis, and age

Mobility was also significantly associated with higher odds of receiving information about HIV prevention (mobility to one location: aOR: 2.11 (95% CI: 1.42, 3.12); mobility to > 1 location: aOR: 2.84 (95% CI: 1.59, 5.08)), and with higher odds of participating with an HIV prevention organization for individuals who were mobile to one location compared to those who were non-mobile (aOR: 2.88 (95% CI: 1.66, 4.99)). Three questions were included in the survey to ascertain the respondent’s HIV-related knowledge. Mobility to one location was associated with increased odds of correctly identifying anal sex as the type of sex with the greatest risk of HIV transmission (aOR: 2.22 (95% CI: 1.17, 4.23)) and correctly stating that HIV can be transmitted through shared needles (aOR: 1.78 (95% CI: 1.17, 2.70)). Mobility to more than one location was associated with decreased odds of answering each of these questions correctly (aOR for the question related to anal sex: 0.28 (95% CI: 0.10, 0.73); aOR for the question related to needle sharing: 0.48 (95% CI: 0.27, 0.85)). Mobility was not found to be significantly associated with stigma, nor with STI symptoms.

As described in the methods section, covariates were selected a priori based on prior literature and the authors’ understanding of HIV and social vulnerabilities. Nevertheless, the McFadden pseudo-R^2^ statistic was consulted to assess the fit of the resulting models. For each model, the inclusion of the selected covariates improved the model fit when compared to the single variable model.

To specifically address the hypothesis that access to services available in the capital city of Bissau may be driving the differences observed for our primary analyses, we conducted secondary analyses considering the participant’s relationship with Bissau (lives outside Bissau and does not travel to Bissau; lives outside Bissau and travels to Bissau, or lives in Bissau) as the exposure of interest. Findings from these analyses can be found in Table 6. For all the results that follow, the reference group was “living outside Bissau and not traveling to Bissau”. Living in Bissau was associated with lower odds of receiving information on HIV prevention (aOR: 0.61 (95% CI: 0.40, 0.95). Both traveling to Bissau and living in Bissau were also associated with lower odds of correctly answering two of the three HIV knowledge questions, including lower odds of correctly identifying anal sex as the type of sex with greatest risk of HIV transmission (traveling to Bissau: aOR: 0.43, 95% CI (0.21, 0.91), living in Bissau: aOR 0.14, 95% CI (0.07, 0.28)) and lower odds of correctly stating that HIV can be transmitted through sharing needles (traveling to Bissau: aOR: 0.55, 95% CI (0.31, 0.97), living in Bissau: aOR 0.48, 95% CI (0.30, 0.77)). As in the primary analysis, the inclusion of potential confounders as covariates improved model fit. However, model fit as assessed by the McFadden pseudo R^2^ statistic was generally not as good for adjusted models in the secondary analysis as for adjusted models in the primary analysis that assessed the extent of mobility. This suggests that the primary analysis (focused on the extent of mobility) better explained the variation in the social and HIV vulnerabilities than the secondary analysis (focused on travel to and residence in Bissau).

**Table 6:** Associations between mobility to and residence in Bissau and HIV vulnerabilities/prevention indicators among FSW in four cities in 2017 and 2018 in Guinea-Bissau (*N* = 567)

Outcome/HIV vulnerability	Unadjusted OR (95% CI)*	*P*-value**	Adjusted*** OR (95% CI)*	*P*-value**
*Sex without a condom*				
With a client (last 3 sex acts)				
Lives outside Bissau, travels to Bissau	2.23 (0.92, 5.44)	0.077	2.44 (0.96, 6.22)	0.061
Lives in Bissau	1.23 (0.60, 2.55)	0.575	1.40 (0.61, 3.22)	0.424
With a non-paying partner (last 3 sex acts)				
Lives outside Bissau, travels to Bissau	1.97 (0.98, 3.96)	0.057	1.66 (0.80, 3.43)	0.172
Lives in Bissau	**2.06 (1.21, 3.49)**	0.007	1.70 (0.94, 3.07)	0.078
*Health and healthcare*				
Visited health center for own health (ever)				
Lives outside Bissau, travels to Bissau	0.96 (0.55, 1.66)	0.871	1.24 (0.68, 2.23)	0.483
Lives in Bissau	1.05 (0.71, 1.55)	0.823	1.01 (0.63, 1.62)	0.957
Reported any STI symptoms (past 12 months)				
Lives outside Bissau, travels to Bissau	1.38 (0.78, 2.43)	0.266	1.67 (0.92, 3.04)	0.092
Lives in Bissau	1.10 (0.72, 1.66)	0.658	1.10 (0.68, 1.80)	0.688
*Opportunities to learn about HIV prevention*				
Received info on HIV prevention (past 6 months)				
Lives outside Bissau, travels to Bissau	1.40 (0.80, 2.44)	0.236	1.37 (0.77, 2.44)	0.28
Lives in Bissau	**0.67 (0.45, 0.98)**	0.037	**0.61 (0.40, 0.95)**	0.029
Participated in activity to promote the rights of FSW (past 6 months)				
Lives outside Bissau, travels to Bissau	0.59 (0.23, 1.55)	0.287	0.81 (0.29, 2.29)	0.691
Lives in Bissau	1.06 (0.59, 1.90)	0.835	1.08 (0.51, 2.27)	0.844
Participated in HIV prevention organization (past 6 months)				
Lives outside Bissau, travels to Bissau	**0.47 (0.23, 0.97)**	0.042	0.57 (0.25, 1.28)	0.173
Lives in Bissau	0.77 (0.49, 1.20)	0.243	0.57 (0.31, 1.04)	0.065
*HIV knowledge*				
Able to correctly identify anal sex as type of sex with greatest risk of HIV transmission				
Lives outside Bissau, travels to Bissau	**0.42 (0.20, 0.86)**	0.017	**0.43 (0.21, 0.91)**	0.027
Lives in Bissau	**0.19 (0.11, 0.33)**	< .001	**0.14 (0.07, 0.28)**	< .001
Able to correctly identify water-based lubricant as safest option with condoms				
Lives outside Bissau, travels to Bissau	0.65 (0.13, 3.29)	0.603	0.57 (0.11, 2.94)	0.497
Lives in Bissau	**3.99 (1.66, 9.59)**	0.002	2.02 (0.77, 5.31)	0.154
Able to correctly state that HIV can be transmitted through sharing needles				
Lives outside Bissau, travels to Bissau	**0.46 (0.26, 0.80)**	0.006	**0.55 (0.31, 0.97)**	0.039
Lives in Bissau	**0.50 (0.33, 0.75)**	0.001	**0.48 (0.30, 0.77)**	0.002
*Stigma*				
Reported experienced stigma (past 6 months)				
Lives outside Bissau, travels to Bissau	1.24 (0.49, 3.12)	0.649	1.45 (0.55, 3.84)	0.45
Lives in Bissau	1.86 (0.97, 3.55)	0.062	1.58 (0.74, 3.37)	0.238
Reported anticipated or experienced healthcare stigma (past 6 months)				
Lives outside Bissau, travels to Bissau	0.65 (0.13, 3.29)	0.603	0.67 (0.13, 3.51)	0.636
Lives in Bissau	1.18 (0.44, 3.12)	0.742	0.94 (0.30, 2.91)	0.911
Reported stigma from family or friends (past 6 months)				
Lives outside Bissau, travels to Bissau	0.54 (0.17, 1.70)	0.294	0.47 (0.15, 1.52)	0.209
Lives in Bissau	0.55 (0.26, 1.16)	0.116	0.47 (0.20, 1.12)	0.088
Reported stigma from police (past 6 months)				
Lives outside Bissau, travels to Bissau	0.29 (0.06, 1.30)	0.105	0.37 (0.08, 1.76)	0.212
Lives in Bissau	0.60 (0.28, 1.27)	0.182	0.74 (0.29, 1.89)	0.525

Unadjusted and adjusted associations between mobility to/residence in Bissau and self-reported social and HIV vulnerabilities among adult female sex workers recruited via respondent-driven sampling by Enda Santé-Bissau in 2017 and 2018 across Bissau, Bissorã, Bafatá, and Gabu in Guinea Bissau, Africa (*N* = 567)

^*^ The reference group for all ORs and aORs is: lives outside Bissau, does not travel to Bissau

^**^ Bold text indicates a significant association at an alpha of < 0.05

^***^ Adjusted for migration, education, self-reported HIV diagnosis, and age

## Discussion

The findings from this study suggest different patterns and dynamics of risk factors for HIV among female sex workers according to their level of mobility. Mobility to more than one destination in the past six months was associated with social and HIV vulnerabilities, most notably substantially higher odds of sex without a condom with both clients and non-paying partners compared to non-mobile individuals. Mobility to just one destination was associated with some detrimental and some protective factors, suggesting a complex dynamic among sex workers with a history of mobility.

Contrary to our hypothesis and existing evidence that mobility results in limited awareness of local services, mobile FSW generally had greater access to services, including higher odds of having received information about HIV prevention and participating in activities with an HIV prevention program than their non-mobile counterparts. This may represent the presence and utilization of programs, either through community-based organizations or healthcare services, with the specific goal of serving mobile sex workers, or it may represent the greater availability of prevention programming and resources for sex workers or for the general population in their destinations. The data may further suggest that services are delivered in a way in which is accessible to this population. Since mobile individuals in this study had higher (though not significantly higher) odds of reporting STI symptoms or diagnosis in the past 12 months, this may also reflect travel for care-seeking. To address the possibility that services in Bissau, specifically, were driving this observation, we secondarily analyzed differences in vulnerabilities associated with respondents’ relationship with Bissau: residing outside Bissau, residing outside Bissau but traveling to Bissau, or residing in Bissau. Contrary to our hypothesis, we found that residing in Bissau was associated with lower odds of receiving HIV prevention information and lower odds of correctly answering two of the three HIV knowledge questions. This could reflect differences in the availability of prevention information in Bissau compared to other locations or the level of engagement with services among the FSW population who reside or travel there.

Mobility was not consistently associated with experiencing stigma from family, the healthcare setting, or police in this study. Although the association between stigma and mobility has been inconsistent across settings and studies, some have observed decreased experiences of stigma among migrant FSW (migrant, as defined in the study, may have some overlaps with mobility as we define it here) [36]. Stigma was reported somewhat less frequently than in a similar survey of FSW in Togo, but the study described here looked at reported stigma in the previous six months, while the study in Togo considered stigma that had ever been experienced [36]. Sex workers with some level of mobility may have the option to seek healthcare services in areas in which they feel more comfortable or experience better treatment. Being mobile may actually facilitate confidentiality in health facilities or in areas in which they may interact with uniformed officers [37]. In addition, mobility may also result in less contact with family members and therefore less exposure to potential stigmas from that network. There is likely a complex relationship between stigma and mobility among sex workers which may be mediated by both behavioral and environmental factors which may influence the potential exposures to stigma.

Although mobility was associated with increased access to and uptake of HIV services, this study also observed that mobility was associated with detrimental outcomes related to HIV risks. Mobility to more than one location among sex workers was associated with condomless sex with both clients and non-paying partners, possibly reflecting a lack of access to condoms among mobile FSW. Mobility to just one location was associated with lower odds of condomless sex with clients. This suggests different dynamics of mobility among individuals mobile to just one location and those mobile to multiple location – perhaps this represents mobility to a single location with which the individual was familiar and had access to resources such as condoms. However, mobility was still associated with higher odds of condomless sex with non-paying partners, suggesting that different factors may influence decisions regarding condom use with clients vs. non-paying partners. Additionally, our secondary analyses found that living in Bissau was associated with more condomless sex with both clients and non-paying partners as compared to living elsewhere and not traveling to Bissau, and that traveling to Bissau is associated with higher odds of condomless sex with non-paying partners. The observation that mobility as well as living in and traveling to Bissau were both associated with more HIV related information but also with higher odds of sex without a condom suggest that HIV prevention services may not be effectively serving these populations.

There were several limitations to these analyses. When applying these results, it should be noted that the context of HIV interventions for this population may have changed since these data were collected: for instance, there may be more recent developments in the availability of PrEP in the region. This study may be subject to selection bias if participants avoided participation due to stigma associated with sex work. Conducting an analysis using data from a survey not specifically designed to investigate mobility comes with a number of challenges and limitations. Since this is a cross-sectional survey, it is also not possible to infer causality based on these data. The time frames in which mobility was reported are not the same as the time frames for which several other variables of interest are reported, it is possible that the observed associations are spurious. Additionally, although the respondent driven sampling method has several strengths (it is designed to reflect the diversity of the population, reach hard-to-reach populations effectively, and was paired with statistical tools to produce population-level estimates of characteristics) it is non-random, and not necessarily a representative sample of all FSW in Guinea-Bissau nor in each survey location. The study may also be subject to information bias if there were systematic errors in reporting of variables. For instance, many respondents listed an income of 0 even though income from sex work was an inclusion criterion for participation, suggesting that this question about income may have been misunderstood by many participants. Finally, mobility was defined based on reports of spending the night outside of the respondent’s place of residence last six months, but this definition and the data available lack specificity about the number and length of these trips. This means that our broad definition of mobility likely encompasses individuals traveling for a number of different reasons and living in a variety of different contexts. Future work should consider more nuanced definitions of mobility that take into account the number and duration of trips to better understand these populations’ experiences.

## Conclusions

This study found that while mobile FSW in Guinea-Bissau are reached by some HIV prevention services, mobility among FSW, particularly mobility to more than one destination, is associated with higher odds of condomless sex. These findings suggest that despite HIV prevention services reaching mobile FSW, such services may not effectively or adequately serve this key population. Future research should consider the complexity of mobility in the FSW population in West Africa and examine the context, frequency, destination, and purpose of mobility, since this work suggests that mobility may have heterogenous effects depending on these and other factors. It should also consider assessing strategies for providing HIV services that are effective for mobile FSW. Although mobile FSW are currently reached by certain preventive services and messaging, highly mobile individuals still experience more HIV-related risks than their non-mobile peers and may not benefit fully from access alone. These analyses highlight the need to provide HIV services that are appropriate and effective for mobile FSW in Guinea-Bissau.

### Supplementary Information

**Additional file 1: Supplementary Table 1.** Variable definitions and survey questions.

## Data Availability

Data and code are available upon reasonable request.

## Abbreviations

aOR: Adjusted odds ratio
CI: Confidence interval
CNES: Comité Nacional de Ética na Saúde
FSW: Female sex workers
HIV: Human immunodeficiency virus
IBBS: Integrated bio-behavioral survey
IQR: Interquartile range
LMIC: Low- or middle-income country
OR: Odds ratio
RDS: Respondent-driven sampling
STI: Sexually transmitted infection
