# Supplementary material for: Mobility and HIV vulnerabilities among female sex workers in Guinea-Bissau: findings from an integrated bio-behavioral survey
Source: BMC Public Health. 2023 Sep 25;23:1856. doi: 10.1186/s12889-023-16744-y (PMC10518914; doi:10.1186/s12889-023-16744-y)
Supplement: Supplementary file 1 — Additional file 1: Supplementary Table 1. Variable definitions and survey questions. [file 12889_2023_16744_MOESM1_ESM.docx]

|  | | Supplementary Table 1: Variable definitions and survey questions | | | |  |
| --- | --- | --- | --- | --- | --- | --- |
| **Variable** | **Relevant survey questions** | | **Variable Type** | **Definition** | **Time period** | **Missingness and assumptions** |
| Mobility | “In the past 6 months, other than your usual place of residence, in how many different cities/towns have you spent the night?” | | Categorical | Non-mobile/mobile to one location/mobile to two or more locations | Last 6 months | Considered non-mobile if participant did not report |
| City of residence | Survey administrator indicated city where survey takes place | | Categorical | Bissau/ Bissorã/Bafatá/Gabu | N/A | No missing values. |
| Income | "On average, what is your monthly income from formal and informal work? (includes both sex work and other activities)" | | Categorical | 0/0-100.000 XOF/>100,000 XOF | N/A | Considered missing if participant refused or did not know. Missing for 11 participants. |
| Education | "What is the highest level of education that you have received?" | | Categorical | None/Quaranic school only or some or completed primary school/some secondary school/completed secondary school or above | N/A | Considered missing if participant refused, did not know, or indicated “other”. Missing for 8 participants. |
| Migration | "In what country were you born?"; "In what region of Guinea-Bissau were you born?" | | Categorical | Non-migrant/domestic migrant (born in different region of Guinea-Bissau than where they currently reside)/international migrant (born outside of Guinea-Bissau) | N/A | No missing values. |
| Self-reported previous HIV diagnosis | "Have you ever been told by a healthcare provider that you have HIV?" | | Binary | Has been told they have HIV/have not been previously told they have HIV, including those who have never been tested | N/A | Considered not to have previous HIV diagnosis if participant did not know or refused to answer. |
| Marital status | “What is your current marital status?” | | Categorical | Never married/married or partnered/divorced or widowed | N/A | No missing values. |
| Employment outside of sex work | “What is your current primary employment status other than sex work?” | | Categorical | No employment or retired/reports employment outside of sex work (formally or informally self employed, public or private employed)/student | N/A | Missing for 2 participants. |
| Tested positive for HIV | N/A – biological test | | Binary | Tested positive for HIV/did not test positive for HIV | Time of study | No missing values. |
| Tested positive for hepatitis B | N/A – biological test | | Binary | Tested positive for hepatitis B/Did not test positive for hepatitis B | Time of study | Missing for one participant. |
| Age | "How old are you?" | | Continuous | Age in years | N/A | No missing values. |
| Years selling sex | “How old are you?”; “At approximately what age did you start providing sexual acts in exchange for money for the first time?” | | Continuous | Current age in years minus age at which participant reports first selling sex | N/A | Considered missing if participant refused or did not know at what age they first sold sex, or if age provided was implausible (ie, 0,1,2,or 3). Missing for 66 participants. |
| Number of children | “How many children are you currently responsible for?” | | Continuous |  | N/A | No missing values. |
| Sex without a condom with client | Participants were asked to report if a condom was used for each of the last three sex acts with a client | | Binary | Sex without a condom/No sex without a condom | Last 3 sex acts | If the participant refused to answer or did not know for any of the three questions, this variable was considered missing. Missing for 46 participants. |
| Sex without a condom with non-paying partner | Participants were asked to report if a condom was used for each of the last three sex acts with a non-paying partner | | Binary | Sex without a condom/No sex without a condom | Last 3 sex acts | If the participant refused to answer or did not know for any of the three questions, this variable was considered missing.  Considered to have not had sex without a condom if participant reported that they had never had sex with a non-paying partner. Missing for 76 participants. |
| Visited health center for own health | “Have you ever gone to a health center for your own health?" | | Binary | Ever/never | Ever | No missing values. |
| Reported STI symptoms | “In the last 12 months, have you had any of the following symptoms of a sexually transmitted infection on or around the vagina or anus?” | | Binary | Reported STI symptoms/did not report any symptoms | Last 12 months | No missing values. |
| PrEP usage | “Have you ever taken ARVs to prevent infection (Pre exposure prophylaxis (PrEP))?” | | Binary | Reported PrEP usage ever/did not report PrEP usage ever | Ever | Considered to not have used PrEP if refused or did not know |
| Received HIV prevention info | “In the last six months, have you received any of the following information on HIV prevention?” Answer options included: “How to prevent HIV infection when having sex with men”; “How often to test for HIV infection”; “What to do for treatment and care if I’m living with HIV” and “Other (specify)”. | | Binary | Reported receiving any information/ did not report receiving information | Last 6 months | No missing values. |
| Participated in activity to promote the rights of FSW | “In general, over the past 6 months, how often have you participated in a meeting, march, rally, or gathering to promote the rights of sex workers?” | | Binary | Ever/never | Last 6 months | No missing values. |
| Participated in HIV prevention organization | “In general, over the past 6 months, how often have you participated in an HIV prevention organization?” | | Binary | Ever/never | Last 6 months | Missing for 2 participants. |
| Able to correctly identify anal sex as type of sex with greatest risk of HIV infection | “What type of sex puts you most at risk for HIV infection?” | | Binary | Answered correctly (“anal sex”)/did not answer correctly | N/A | Respondents were not prompted with a list of options. If not answered, considered to not be able to correctly identify. |
| Able to correctly identify water-based lubricant as safest option | “Which is the safest lubricant to use during anal or vaginal sex with latex condoms?” | | Binary | Answered correctly (“water-based lubricant”)/did not answer correctly | N/A | Respondents were not prompted with a list of options. If not answered, considered to not be able to correctly identify. |
| Able to correctly state that HIV can be transmitted through sharing needles | “Can you get HIV from a needle that someone else has already used to inject a drug or medicine?” | | Binary | Answered correctly (“yes”)/did not answer correctly | N/A | Respondents were not prompted with a list of options. If not answered, considered to not be able to correctly identify. |
| Experienced stigma | "Has a uniformed officer ever harassed (verbally or physically) or intimidated you because you sell sex?"; "Have you ever been arrested, and was this arrest on charges related to sex work?"; "Has someone ever verbally harassed you because you sell sex?"; "Has someone ever blackmailed you because you sell sex?"; "Has someone ever physically harassed or hurt you because you sell sex?"; "Has someone ever forced you to have any type of sex when you did not want to?" and "Do you believe any of these experiences of forced sex were related to the fact that you sell sex?" | | Binary | Answered affirmatively to any/did not answer affirmatively to any.  Please see Grosso et al, 2019 | Last 6 months | Considered to have not experienced stigma if one or more questions were missing and others were answered “no”. Considered missing only if all relevant survey questions were missing. |
| Anticipated or experienced healthcare stigma | "Have you ever felt afraid seek health services because you worried someone may learn you sell sex?"; "Have you ever avoided seeking health services because you worried someone may learn you sell sex?"; "Have you ever been denied health services or had someone keep you from receiving health services because you sell sex?"; "Have you ever heard health care providers make discriminatory remarks or gossip about you because you sell sex?"; " Have you ever felt that you were not treated well in a health center because you sell sex?"; | | Binary | Answered affirmatively to any/did not answer affirmatively to any | Last 6 months | See “experienced stigma” |
| Stigma from family or friends | "Have you ever felt excluded from family activities because you sell sex?"; "Have you ever felt that family members have made discriminatory remarks or gossiped about you because you sell sex?"; "Have you ever felt rejected by your non sex worker friends because you sell sex?" | | Binary | Answered affirmatively to any/did not answer affirmatively to any | Last 6 months | See “experienced stigma” |
| Stigma from police | "Have you ever avoided carrying condoms because you were afraid that they might get you in trouble with a uniformed officer?"; " Have you ever seen or heard about a uniformed officer confiscating or destroying condoms held by a sex worker (including you) or an outreach worker?"; "Have you ever felt that a uniformed officer (i.e. the police, military, security officer, etc.) refused to protect you because you sell sex?" | | Binary | Answered affirmatively to any/did not answer affirmatively to any | Last 6 months | See “experienced stigma” |
